# Supplementary material for: Comparative Analysis of the Physicochemical and Biological Characteristics of Freeze-Dried PEGylated Cationic Solid Lipid Nanoparticles
Source: Pharmaceuticals (Basel). 2023 Nov 9;16(11):1583. doi: 10.3390/ph16111583 (PMC10675625; doi:10.3390/ph16111583)
Supplement: Supplementary file 1 [file pharmaceuticals-16-01583-s001.zip › pharmaceuticals-2670274-supplementary.pdf]

Supplementary material

Table S1. Measurements of the glass transition temperature (Tg) of each PEG formulation developed.

PEG (150 mg)	Tg (°C)
Brij S100	-35.57
Gelucire	-21.15
Myrj 52	-25.62
Myrj S100	-38.18

Table S2. Correlations and regressions between the physicochemical characteristics (PdI, PSD, and ZP) of suspended cSLNs and the elapsed time (30 days) of each PEG formulation at two temperatures (4 °C and 25 °C) exposed.

	PdI				PSD (nm)				ZP (mV)			
PEG (150 mg)	4 °C		25 °C		4 °C		25 °C		4 °C		25 °C	
Brij S100	$P = 0.007$	$R^2 = 0.731$	$P < 0.001$	$R^2 = 0.888$	$P = 0.013$	$R^2 = 0.671$	$P = 0.008$	$R^2 = 0.719$	$P = 0.266$	$R^2 = 0.200$	$P = 0.520$	$R^2 = 0.072$
Gelucire	$P = 0.509$	$R^2 = 0.074$	$P = 0.001$	$R^2 = 0.864$	$P = 0.023$	$R^2 = 0.604$	$P = 0.071$	$R^2 = 0.450$	$P = 0.030$	$R^2 = 0.572$	$P = 0.160$	$R^2 = 0.300$
Myrj 52	$P = 0.688$	$R^2 = 0.029$	$P = 0.005$	$R^2 = 0.755$	$P = 0.032$	$R^2 = 0.561$	$P = 0.034$	$R^2 = 0.556$	$P = 0.007$	$R^2 = 0.728$	$P = 0.067$	$R^2 = 0.453$
Myrj S100	$P = 0.653$	$R^2 = 0.036$	$P = 0.022$	$R^2 = 0.609$	$P = 0.040$	$R^2 = 0.533$	$P = 0.001$	$R^2 = 0.844$	$P < 0.001$	$R^2 = 0.952$	$P = 0.134$	$R^2 = 0.334$

Table S3. Correlations and regressions between the physicochemical characteristics (PdI, PSD, and ZP) of lyophilized cSLNs and the elapsed time (one year) of each PEG formulation at two temperatures (4 °C and 25 °C) exposed.

	PdI				PSD (nm)				ZP (mV)			
PEG (150 mg)	4 °C		25 °C		4 °C		25 °C		4 °C		25 °C	
Brij S100	$P = 0.652$	$R^2 = 0.056$	$P = 0.104$	$R^2 = 0.523$	$P = 0.024$	$R^2 = 0.758$	$P = 0.088$	$R^2 = 0.559$	$P = 0.039$	$R^2 = 0.695$	$P = 0.023$	$R^2 = 0.763$
DSPE	$P = 0.607$	$R^2 = 0.072$	$P = 0.752$	$R^2 = 0.028$	$P = 0.118$	$R^2 = 0.496$	$P = 0.303$	$R^2 = 0.258$	$P = 0.469$	$R^2 = 0.138$	$P = 0.774$	$R^2 = 0.023$
Gelucire	$P = 0.040$	$R^2 = 0.691$	$P = 0.323$	$R^2 = 0.241$	$P = 0.080$	$R^2 = 0.576$	$P = 0.867$	$R^2 = 0.008$	$P = 0.655$	$R^2 = 0.055$	$P = 0.238$	$R^2 = 0.325$
Myrj 52	$P = 0.013$	$R^2 = 0.819$	$P = 0.139$	$R^2 = 0.460$	$P = 0.916$	$R^2 = 0.003$	$P = 0.286$	$R^2 = 0.275$	$P = 0.628$	$R^2 = 0.064$	$P = 0.694$	$R^2 = 0.043$
Myrj S100	$P = 0.450$	$R^2 = 0.149$	$P = 0.173$	$R^2 = 0.406$	$P = 0.065$	$R^2 = 0.615$	$P = 0.159$	$R^2 = 0.428$	$P = 0.005$	$R^2 = 0.374$	$P = 0.922$	$R^2 = 0.003$

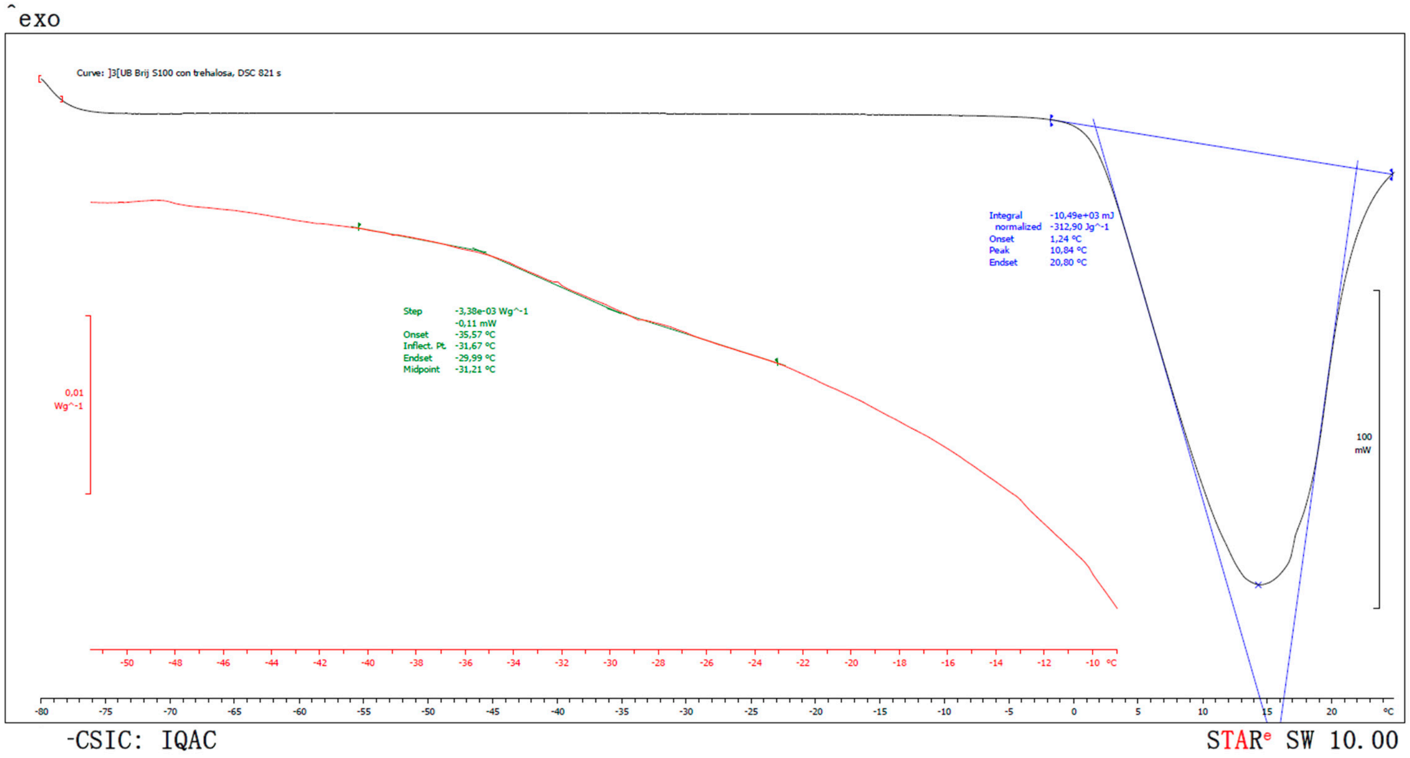
^exo
Curve:]3[UB Brij S100 con trehalosa, DSC 821 s
Integral -10,49e+03 mJ
normalized -312,90 Jg^-1
Onset 1,24 °C
Peak 10,84 °C
Endset 20,80 °C
Step -3,38e-03 Wg^-1
-0,11 mW
Onset -35,57 °C
Inflect. Pt. -31,67 °C
Endset -29,99 °C
Midpoint -31,21 °C
0,01 Wg^-1
100 mW
-CSIC: IQAC
STARe SW 10.00

A

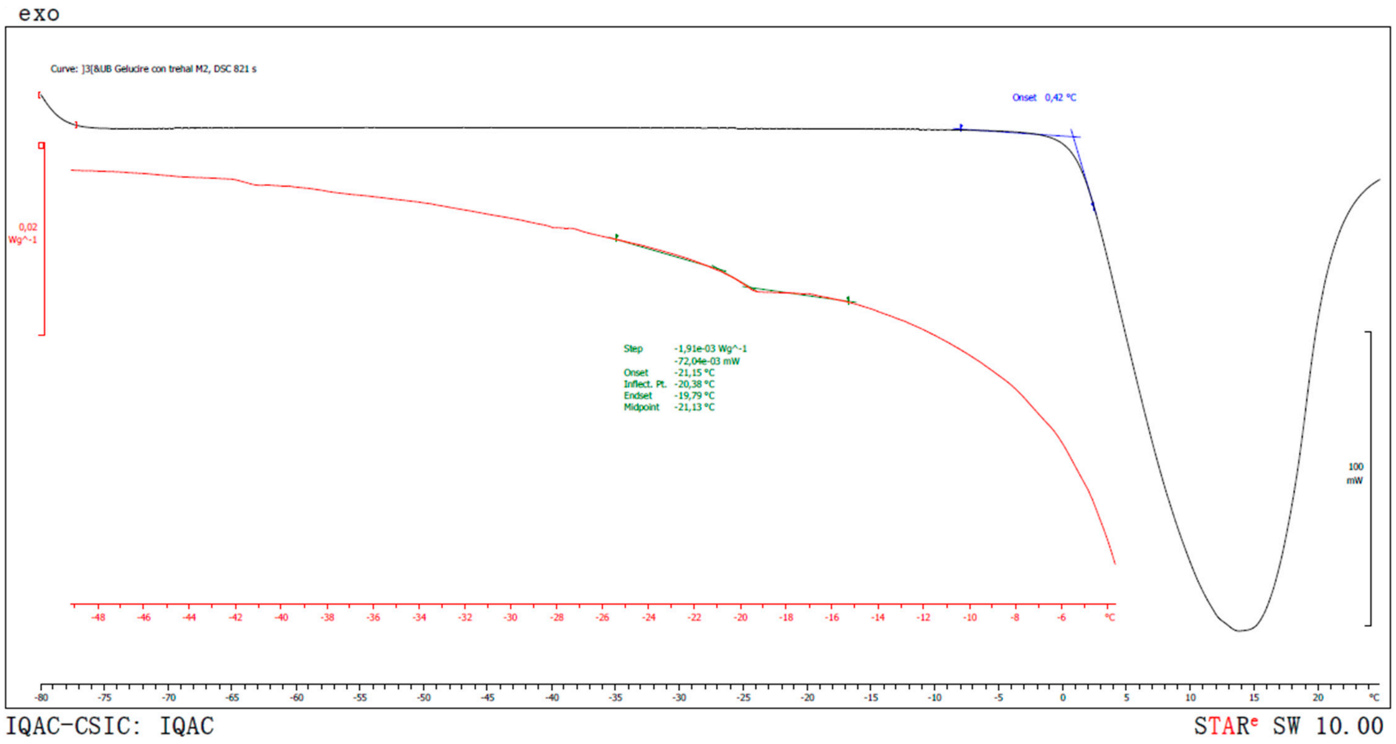
exo
Curve:]3[&UB Gelucire con trehal M2, DSC 821 s
Onset 0,42 °C
0,02 Wg^-1
Step -1,91e-03 Wg^-1
-72,04e-03 mW
Onset -21,15 °C
Inflect. Pt. -20,38 °C
Endset -19,79 °C
Midpoint -21,13 °C
100 mW
IQAC-CSIC: IQAC
STARe SW 10.00

B

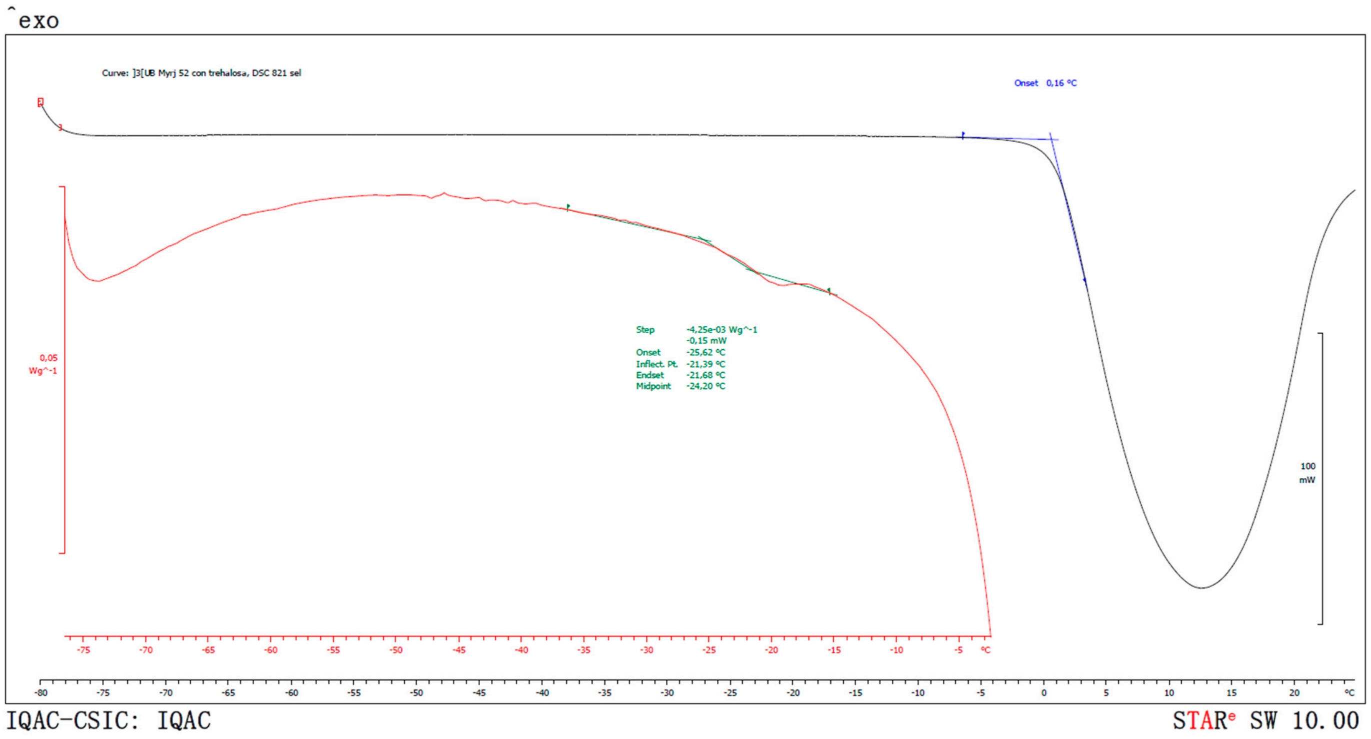
^exo
Curve:]3[UB Myrj 52 con trehalosa, DSC 821 sel
Onset 0,16 °C
Step -4,25e-03 Wg^-1
-0,15 mW
Onset -25,62 °C
Inflect. Pt. -21,39 °C
Endset -21,68 °C
Midpoint -24,20 °C
0,05 Wg^-1
100 mW
-75 -70 -65 -60 -55 -50 -45 -40 -35 -30 -25 -20 -15 -10 -5 °C
-80 -75 -70 -65 -60 -55 -50 -45 -40 -35 -30 -25 -20 -15 -10 -5 0 5 10 15 20 °C
IQAC-CSIC: IQAC
STARe SW 10.00

C

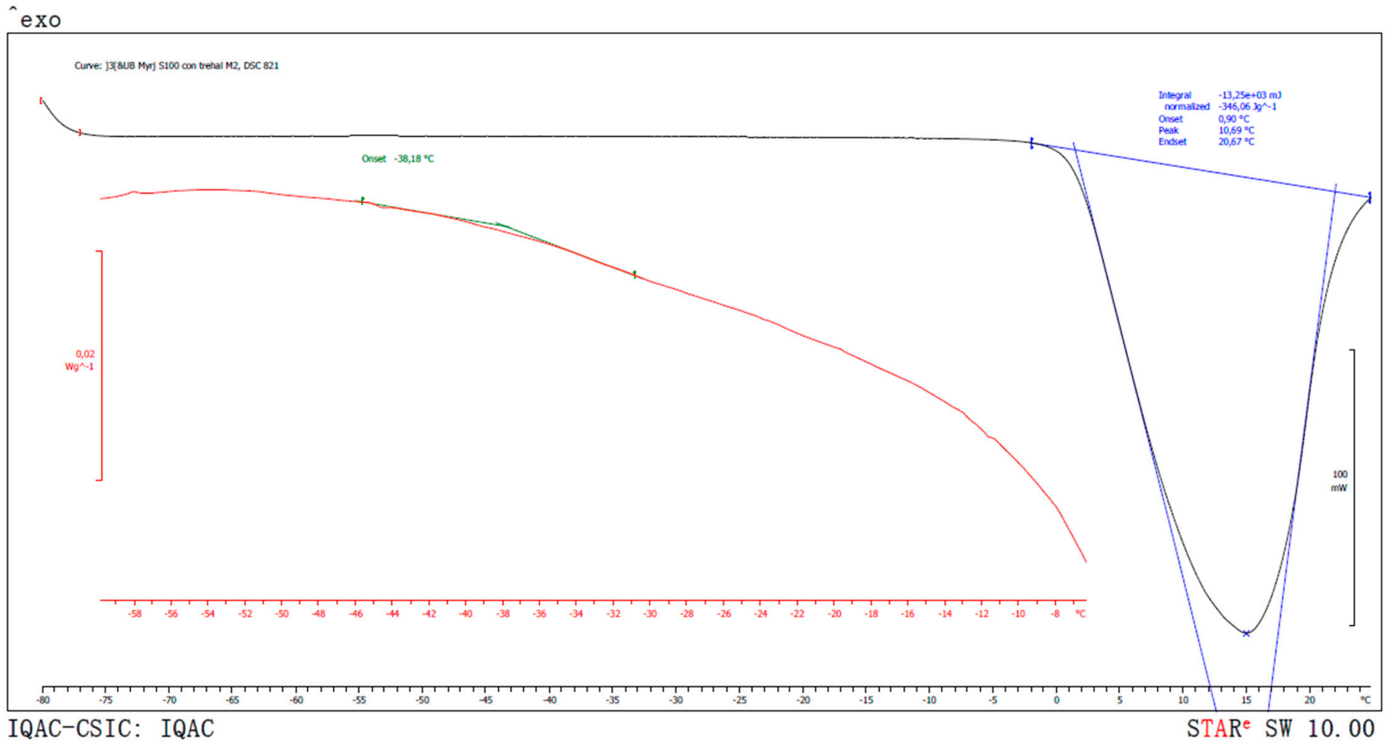
^exo
Curve:]3[&UB Myrj S100 con trehal M2, DSC 821
Integral -13,25e+03 mJ
normalized -346,06 Jg^-1
Onset 0,90 °C
Peak 10,69 °C
Endset 20,67 °C
Onset -38,18 °C
0,02 Wg^-1
100 mW
-58 -56 -54 -52 -50 -48 -46 -44 -42 -40 -38 -36 -34 -32 -30 -28 -26 -24 -22 -20 -18 -16 -14 -12 -10 -8 °C
-80 -75 -70 -65 -60 -55 -50 -45 -40 -35 -30 -25 -20 -15 -10 -5 0 5 10 15 20 °C
IQAC-CSIC: IQAC
STARe SW 10.00

D

Figure S1: DSC analysis of the four PEG-cSLNs suspended in a solution with 5% of trehalose. Brij S100 (**A**), Gelucire (**B**), Myrj 52 (**C**), and Myrj S100 (**D**).
